# Supramolecular Binding with Lectins: A New Route for Non-Covalent Functionalization of Polysaccharide Matrices

**DOI:** 10.3390/molecules27175633

**Published:** 2022-09-01

**Authors:** Devis Montroni, Matteo Di Giosia, Matteo Calvaresi, Giuseppe Falini

**Affiliations:** Department of Chemistry “Giacomo Ciamician”, Alma Mater Studiorum-University of Bologna, Via Selmi 2, 40126 Bologna, Italy

**Keywords:** chitin, polysaccharide, lectin, WGA, functionalization, supramolecular

## Abstract

The chemical functionalization of polysaccharides to obtain functional materials has been of great interest in the last decades. This traditional synthetic approach has drawbacks, such as changing the crystallinity of the material or altering its morphology or texture. These modifications are crucial when a biogenic matrix is exploited for its hierarchical structure. In this work, the use of lectins and carbohydrate-binding proteins as supramolecular linkers for polysaccharide functionalization is proposed. As proof of concept, a deproteinized squid pen, a hierarchically-organized β-chitin matrix, was functionalized using a dye (FITC) labeled lectin; the lectin used was the wheat germ agglutinin (WGA). It has been observed that the binding of this functionalized protein homogenously introduces a new property (fluorescence) into the β-chitin matrix without altering its crystallographic and hierarchical structure. The supramolecular functionalization of polysaccharides with protein/lectin molecules opens up new routes for the chemical modification of polysaccharides. This novel approach can be of interest in various scientific fields, overcoming the synthetic limits that have hitherto hindered the technological exploitation of polysaccharides-based materials.

## 1. Introduction

Polysaccharides are biopolymers widely diffused in nature; cellulose and chitin are the two most abundant biopolymers on earth [1,2,3,4,5]. The properties of polysaccharides and their biological roles are diverse, i.e., involving signaling, water retention, mechanical protection, and structural integrity [6,7,8]. In particular in the last two roles, polysaccharides with large varieties of different functional morphologies have been observed [9,10,11,12]. From this view, chitin is likely the biopolymer with the most diverse varieties of hierarchical structures [13,14,15,16,17,18]. It has been documented that these structures exhibit greater mechanical resistance, positively influence biomineralization processes, and modulate the mechanical stress distribution and the propagation of light and cracks [14,15,17,19,20,21,22].

The abundance of polysaccharides, their diverse properties, and their availability as highly-organized structures have led to significant interest in their technological applications. Indeed, there is a vast amount of literature available on this topic, which includes a wide range of fields, such as cosmetics, medicine, construction, water remediation, etc. [23,24,25,26,27,28,29,30,31,32,33,34,35]. Among these, chitin has received special attention for its high mechanical resistance, biocompatibility, biodegradability, crystallinity, mild antimicrobial activity, transparency, and abundance of highly-organized biogenic matrices [23,36,37,38,39,40,41].

In many of these studies, chitin, similar to many other polysaccharides, was provided with new properties by chemical functionalization to obtain innovative functional materials [42,43,44,45]. Covalent functionalization is the most commonly used method of conferring new properties to a material. Despite the high versatility and stability of the covalent bioconjugation approach, such an approach usually affects the chemical structure of the polymer and its preexisting order, such as its morphology or crystallinity [46]. This is a major concern when a hierarchically-organized biogenic matrix is used with the intent to exploit its remarkable micro- and nanoscale structural properties. For example, functionalization on chitin usually targets the amide group hydrolyzed into an amine, which means that a step introducing positive charges in the material is performed [47,48,49,50]. This deacetylation step was observed to induce significant changes in the mechanical resistance of the material, even when performed on just 17% of the monomers (decreasing more than 10 times the Young’s modulus of the material) [51]. Higher degrees of deacetylation can also induce changes in the crystallographic structure [52]. Moreover, the functionalization of polysaccharide-based organized matrices necessarily implies syntheses in the hetero-phase. Despite the multiple advantages provided by hetero-phase syntheses, such as the easy purification processes of unconjugated molecules and the possible synthesis subproducts, the different diffusion rates of a reactive molecule into a multiscale porous matrix may preclude the uniform modification of the targeted chemical moieties. As a result, a pore-size-dependent functionalization degree could occur, producing non-homogeneous functional materials.

Lectins are a group of proteins, typical of plants, capable of specific recognition, and reversible binding to carbohydrate moieties without altering the covalent structure of the ligand [53]. These proteins play a major role in biological recognition. In plants, chitin-binding lectins are applied in the defense against pests and parasites [54,55,56]. Due to their specificity and strong binding, lectins have been widely applied in diagnostics [57,58], and, less frequently, in drug delivery [53,59]. On the other hand, their use in material science has been neglectable; there have only been a few studies aimed at enhancing cell adhesion through protein–carbohydrate interactions [60,61].

A wide variety of lectins with a specific affinity for polysaccharides is available [62]. Of these, wheat germ agglutinin (WGA) is one of the most studied. This protein is a homodimer composed of four homologous domains with a total of eight potential binding sites and a molecular weight of 36 kDa [63,64]. It has a strong binding capability and specificity for N-acetylglucosamine oligomers, which increase for sequences above three sugar units, such as chitin [64].

In this work, we propose the use of functionalized lectins as a tool for polysaccharide functionalization. Since the binding with lectins is based on non-covalent interaction [65,66], the hypothesis is that the pre-existing organization and order of the matrix should also be preserved upon binding. Unlike the reactive molecules commonly used for bioconjugation, WGA (as well as many other lectins) is chemically inert, preventing uncontrolled functionalization. Here, compared to the standard heterogenous bioconjugation approach, a different process occurs. In fact, surface functionalization takes place under target-oriented adsorption control [66], where the different diffusion processes of the proteins do not affect the surface coverage of the matrix. Moreover, lectins are multivalent ligands showing enhanced binding capabilities compared to monovalent systems [67]. This is due to a cooperativity effect often attributed to a favorable spatial pre-organization of the ligands or to “rebinding”, where as soon as a single ligand dissociates, another ligand binds in its place. Another advantage of using proteins is that they display a wide number of functional groups, compared to the relatively simple chemical structure of a polysaccharide. Lectins are a suitable platform for non-covalent chitin modification, by following standard procedures commonly used for protein modification in the homo-phase, offering a wide number of chemical targets (carboxylic, amine, thiol, phenolic groups, etc.) for bioconjugation [68].

This study was carried out on a chitin matrix since chitin is one of the most reported polysaccharides in the literature but its functionalization can be problematic due to its insolubility [46,51]. WGA was used since it is the most studied chitin-binding lectin. As a proof of concept, WGA was covalently functionalized with a dye (namely fluorescein-5-isothiocyanate, FITC), allowing for easy detectability of the protein during and after the binding process, by UV-Vis spectrometry. A deproteinized squid pen was chosen as the model of the hierarchically-organized biogenic matrix. Squid pen architecture is well studied, and its aligned nano-fibrillar structure allows for an easy evaluation of the influence of functionalization on the structure at different dimensional scales [69,70].

To summarize, this study presents a new approach for the exploitation of polysaccharide-based organized biogenic matrices and synthetic materials, allowing a one-pot functionalization of the material in an aqueous solution without altering pre-existing ordered hierarchical structures.

## 2. Results

The WGA conjugation with FITC had a 33% reaction yield (percentage of FITC conjugated to WGA in the reaction conditions) that was determined by the UV-Vis analysis, quantifying the number of fluorescein molecules conjugated to WGA (Figure S1). Based on the extinction coefficient of the fluorophore at 495 nm, an average FITC of 3.1 was determined to be conjugated to each WGA. The electrophoretic characterization (carried out on a 15 wt.% SDS-PAGE) revealed the main spot of WGA monomers around 17 kDa (Figure S2). A broader band, characterized by an MW slightly higher than WGA, showed a distribution of proteins conjugated with different FITC numbers. The faint band at 36 kDa for WGA, and the associated bands at higher MWs, corresponded to the residual dimeric form of the protein. Under UV irradiation, the only fluorescent spot came from the WGA-FITC, further confirming the successful conjugation after the purification process.

**Figure 1:**
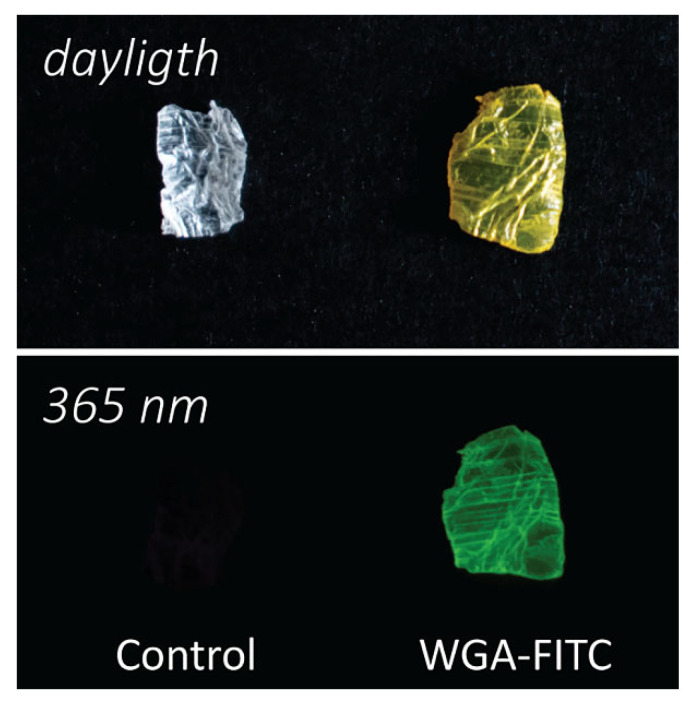
Camera images of the matrices. Camera picture of a chitin sample (control, **left**) and a WGA-FITC treated chitin sample (**right**) viewed in daylight (**top**) and under a UV lamp (365 nm; **bottom**), each sample is about 5 mm large.

The hierarchically-organized chitin-based matrix was obtained from the blade of the squid pen of *Loligo vulgaris* after deproteination by the alkaline treatment [17,46,51,71]. This treatment resulted in a final degree of acetylation of about 88% (acetyl groups on sugar monomer number) [51]. Before exposing the matrix to the lectin, it was swelled in an HCl solution at pH 2, a treatment observed to increase the swelling of β-chitin matrices. This swelling was maintained by moving the matrix to higher pH solutions [17,72]. The matrix was then treated with a WGA-FITC solution 0.5 mg·mL^−1^ with a protein/chitin ratio of 5 wt.% for 72 h in a buffered environment at pH 7.4. In the end, the WGA-FITC bonded to the matrix was 3.3 ± 0.4 wt.% of WGA-FITC, corresponding to 65 ± 9% of the protein in the initial loading solution. The functionalized matrix appeared bright yellow in daylight and showed fluorescence under UV light, Figure 1. A control using pristine WGA, not functionalized with FITC, was carried out to evaluate eventual adsorption differences due to the FITC functionalization. The binding was assessed using the 280 nm absorption band of the protein. No significant difference was found, being 70 ± 10% of the WGA absorbed in the same experimental condition.

**Figure 2:**
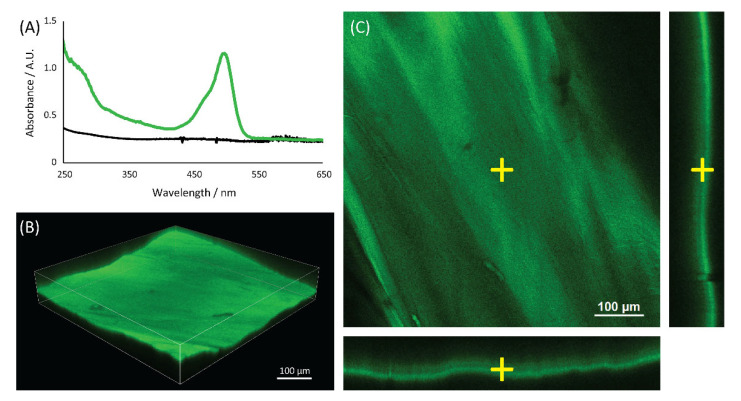
UV-Vis spectroscopic analyses of the matrix. (**A**) UV-Vis spectra of the blank control (black line) and the WGA-FITC treated (green line) chitin samples. The confocal analyses of a wet WGA-FITC treated sample are shown, and both the (**B**) 3D model and (**C**) sections along different planes are reported.

The UV-Vis spectra (Figure 2) of the chitin matrix treated with WGA-FITC showed both the FITC absorption band at 495 nm and the WGA absorption band at 280 nm, while the blank control of the chitin matrix showed no absorption bands. The 495 nm signal of the FITC was used to map the distribution of the WGA-FITC bioconjugates in the chitin matrix using confocal microscopy, Figure 2. The mapping showed a homogeneous distribution of the WGA-FITC bioconjugates along the surface and the bulk of the chitin matrix. No signal was observed in the chitin blank control, Figure S3.

**Figure 3:**
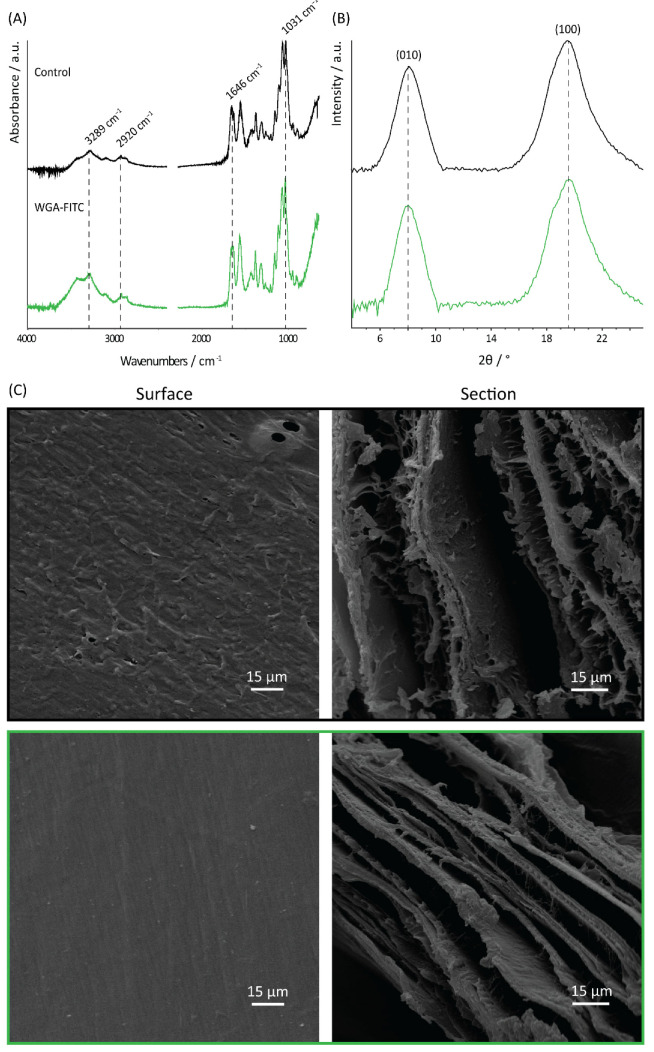
Structural and morphological analyses of the matrix. (**A**) FTIR, (**B**) XRD, and (**C**) SEM analyses of the blank chitin control (black line) and the WGA-FITC-treated chitin samples (green line). Surface image magnification is 800X and the cross-section image magnification is 1000X.

The FTIR and XRD investigation of the functionalized matrix (WGA-FITC treated) showed no differences compared to the blank control, confirming the β-chitin polymorph (Figure 3). Indeed, the FTIR spectra of the pure chitin and the WGA-treated matrix showed the same absorption bands, with no significant shift observable. Among them, the main absorption bands observed were: 1031 cm^−1^, 1068 cm^−1^, 1111 cm^−1^, and 1154 cm^−1^ associated with the vibration modes of the sugar ring; 1262 cm^−1^, 1555 cm^−1^, and 1646 cm^−1^, respectively, amides III, II, and I; 2856 cm^−1^, and 2920 cm^−1^ associated with the CH_3_ and CH_2_ stretching modes; 3289 cm^−1^, and 3424 cm^−1^ associated with N-H and O-H stretching [73,74,75]. A full description of the absorption bands of the two matrices can be found in Table S1. The ratio between the 1646 cm^−1^ (which is associated with both chitin and WGA) and the 1031 cm^−1^ (only associated with chitin) absorption bands was calculated to evaluate the protein binding [76]. A value of 0.46 was calculated for the control sample, while a value of 0.67 was observed for the WGA-treated sample.

In addition, the XRD peak position was not affected by the functionalization, Table 1. The diffraction peak intensity ratio (010)/(100), used as an index of the fibril coherent orientation along the main axis of the pen, and the full width at half maximum (FWHM) of both peaks, used as an index of crystallinity, had no significant difference (*t*-test, *p* = 0.05) between the functionalized sample and the chitin control, Table 1.

SEM observation of both the surface and the section of the sample revealed no significant difference in the morphology, Figure 3. Both the layered structure of the section and the uniaxial fibril/fiber orientation on the matrix surface were maintained. Compared to the control, the functionalized sample appeared to exhibit a smoother surface.

Uniaxial tensile tests were performed on samples treated using WGA not functionalized with FITC; Table 1 and Figure S4. The samples were tested by stretching the squid pen blades along their main axis, which is parallel to the fibrillar orientation. No significant differences (*t*-test, *p* = 0.05) were observed between the control and the WGA-treated chitin matrix.

The desorption of WGA-FITC from the chitin matrix was evaluated by setting the matrix (1–2 mg) in 1 mL of PBS and observing the evolution of the FITC signal in the solution over time. The kinetic was studied for three days; Figure S5. During this time, the WGA-FITC slowly desorbed. After one day, 1.8 ± 0.8% of the WGA-FITC adsorbed to the chitin matrix (0.06 ± 0.04 wt.% of WGA-FITC/chitin) was desorbed. After three days, a desorption of 4 ± 2% of the WGA-FITC (0.13 ± 0.09 wt.% of WGA-FITC/chitin) was observed. At the end of the kinetic experiment, the cuvette was kept in a dark room for ten days and then its UV-Vis spectrum was collected again. The desorption was 7 ± 3% (0.2 ± 0.1 wt.% of WGA-FITC/chitin. No detectable desorption of UV-Vis absorbing matter was observed from the control sample.

## 3. Discussion

Although chemical functionalization is able to introduce new properties to a material, the alteration of the chemical structure of a polymer has a strong influence on its properties, even if a low degree of functionalization is achieved [46,51]. This might be due to changes in the polymer network of interaction, such as hydrogen bond pairing or changes in the crystallographic packing. For example, in Montroni et al. (2021), a drastic change in the swelling of a chitin matrix was observed after the functionalization with a catechol group despite the very low degree of functionalization [46]. Alternatively, the functional group might change the hydrophobicity or polarity at the polymeric chain interface, or insert charges in the materials, inducing electrostatic repulsion. As an example, Gallego et al. reported a functionalization of chitin with 1,6-hexamethylene diisocyanate below 25% (on C6, C3, and C2) showing drastic changes in the solubility, rheology, and thermal properties of the polysaccharide [78]. This issue is particularly relevant when a hierarchically-organized matrix is used with the aim of maintaining its native organization, such as in photonic or porous structures. The introduction of new functionalities and the alteration of the main chemical backbone of the polymer might lead to an alteration of the texture, morphology, or architecture of the matrix. In Machalowski et al., the functionalization with silver nanoparticles of a porous chitinous sponge skeleton is reported to induce a strong reduction of the polymer crystallinity, with a loss of the (020) diffraction peak [79]. This alteration may result in a decrease in the mechanical properties of the matrix.

To overcome the above criticisms, this study proposes a new route for the supramolecular functionalization of polysaccharide matrices using lectins as linkers of functional moieties. Considering the high specificities of lectins, this protocol of functionalization is highly selective for a specific polysaccharide, granting a high specificity even if a composite with a material having analog functional groups is used.

As a representative example of this approach, the deproteinized blade of the squid pen of *L. vulgaris* was functionalized, by supramolecular interaction, with a WGA protein tagged with FITC.

The WGA-FITC bioconjugate bound to the chitinous matrix in the same way as wild type WGA, meaning that the chemical functionalization of the protein did not affect the carbohydrate-binding sites of the lectin. Moreover, the confocal microscopy analysis showed that the WGA-FITC-binding was uniform across the entire matrix. This means that the protein was able to diffuse properly in the inner layers of the squid pen.

The analysis of the FTIR data indicates that no changes in the hydrogen bond network occurred during the functionalization process. In fact, the only alteration observed in the FTIR spectra was the intensity increase of the amide band signals due to the addition of the WGA. The same pristine organization was observed on the crystal structure using XRD, with no visible alteration of crystallinity. This is very important since β-chitin, contrary to α-chitin, presents a (011) crystal plane with no inter-chain hydrogen bonds [80]. In this plane, the interactions are mediated by water molecules in the hydrated form [81,82]. Due to this lack of interaction, this crystal plane is more prone to chemical functionalization, easily exposing the crystal structure to potential alteration. A conserved crystal structure means that the proposed protocol does not affect the crystallographic packing of the biopolymer in any way.

The evaluation of the hierarchical organization of the matrix, performed by XRD peak intensity and profile analyses, showed that the WGA did not introduce modifications, such as misalignments, at the nano-fibrillar level. Furthermore, the SEM images showed that the micro-organization of the chitin fibril, i.e., fibril alignment and lamellar structure, did not change with the functionalization. The smoother surface observed on the functionalized chitin was likely an effect of the homogenous coverage of the matrix with the protein. Apart from this, a clustering of the lamellae is usually visible when a crosslinking is present [46]. This means that the lectin functionalization does not introduce any long-range interaction or cross-linking in the matrix, despite each WGA presenting more than one chitin-binding site.

The absence of cross-linking or other alterations at shorter distances, as in the fibrillar packing, was also confirmed by the mechanical behaviors of the matrices. No significant changes were observed in the mechanical parameters studied. This excludes both the formation of inter-chain cross-linking bridges [83,84,85] and the decrease of the interaction among fibrils, which would significantly alter the mechanical properties of the material, even when present to a low extent [17].

To evaluate the stability of the functionalized chitin, a desorption kinetic was performed. The results showed that in a wet environment the WGA-FITC slowly desorbed from the matrix. After 10 days, the amount desorbed was about 7% of the total WGA-FITC present in the sample. This desorption was very slow (a plateau was not observed in three days) and gradually slowed down over time. This result shows how (after ten days) 93% of the WGA-FITC functionalization was still bound to the matrix in a wet environment. This is remarkable considering the concentration gradient between the matrix and the solution. On the other hand, the slow-release kinetics could find important applications in drug delivery. Since the functionalization is highly specific and can be performed in water over a wide pH range (including physiological conditions) [64,86], the material can be reloaded when necessary, or even modified with a different functionalization depending on the envisioned application.

## 4. Materials and Methods

### 4.1. Materials

Pure wheat germ agglutinin (WGA), from *Triticum vulgare*, was purchased from EY Laboratories, Inc. (San Mateo, CA, USA) and stored in a freezer at −20 °C.

Squid pens from *Loligo vulgaris* were collected from a local market (Bologna, Italy). Once hydrated, the lateral blades were isolated, cleaned with abundant distilled water (carefully eliminating eventual residual tissues), ethanol 70 vol.%, distilled water to remove the ethanol, and then stored dry in a desiccator.

The β-chitin matrices were obtained by putting about 25 g of squid pen in 1 L of boiling 1 M NaOH solution and stirring for 1 h [17,51]. Then, the solution was replaced with a fresh one and stirred under reflux for one hour more (counting the time from when the reflux restarted). The obtained β-chitin was washed two times with a 1 M NaOH solution, and then with distilled water until the wash water was neutral. The chitin obtained was stored dry in a desiccator.

All remaining reagents mentioned were purchased from Merck and used without any further purification.

### 4.2. WGA-FITC Synthesis

FITC bioconjugation to WGA was performed following the standard procedure provided by the supplier. Stock solutions of FITC and WGA were prepared by dissolving (i) 1 mg of FITC in 1 mL of DMSO and (ii) 2 mg of WGA in 1 mL of a 0.1 M sodium carbonate buffer at pH 9. Then, 50 µL of a FITC solution was slowly added to the 1 mL WGA solution in a dropwise manner under gentle stirring. The mixture, contained in an Eppendorf tube, was incubated at 25 °C under constant shaking for 8 h at 700 rpm (ThermoMixer HC, S8012-0000; STARLAB, Hamburg, Germany). Successively, the excess of FITC was removed by two consecutive dialysis cycles in 10 mM of carbonate buffer at pH 9 while the last cycle was carried out in PBS. All dialysis cycles were performed at room temperature (25 °C).

### 4.3. Chitin Functionalization

A chitin sample, cut into a 5 mm side square (dry weight from 4 to 7 mg), was pre-swelled in an HCl solution at pH 2 for 48 h. After the swelling, the sample was soaked in distilled water for 1.5 h, replacing the water every 30 min, and set on a rocking table to eliminate the HCl from the chitin. The sample was then soaked in a 0.5 mg·mL^−1^ lectin solution in a 50 mM phosphate buffer at pH 7.4. The volume of the solution used gave a final WGA/chitin ratio of 5 wt.%. The mixture was shaken on a rocking table for 72 h at room temperature (25 °C) in a dark room. A control experiment was carried out without adding protein to the solution. The sample was then washed three times with 150 µL of the buffer, soaking the sample for 30 min each time, then dried in a desiccator and stored at 4 °C. Once exposed to the protein, all the passages were done keeping the sample in a dark environment.

### 4.4. WGA Adsorption and Desorption Analyses

The WGA adsorption on the chitin matrix was determined by the differences between the initial and final protein amounts in the solution during the chitin functionalization. The protein quantification was determined by evaluating the UV-Vis absorption bands of the FITC at 495 nm (for the WGA-FITC) or of the protein at 280 nm (for WGA). The initial protein solution was analyzed prior to chitin exposure. The final protein amount was determined by analyzing the final solution, after 72 h of exposure to chitin, and added to the buffer aliquots used to wash the functionalized chitin. The protein absorption data were calculated as the average of four independent measurements. Each time, the control experiment was performed without adding the protein to the loading solution.

WGA desorption from the functionalized chitin was evaluated by UV-Vis spectroscopy monitoring the FITC absorption band at 495 nm. A dry functionalized chitin sample (1–2 mg) was set in a plastic cuvette with 1 mL of phosphate saline buffer (PBS). The solution was analyzed every 20 min for 2 h and then every 30 min for three days using a Cary60 spectrophotometer, equipped with the multicell holder (Agilent Technologies), collecting the spectra from 800 to 230 nm. The cuvette was then set in a dark room and analyzed again after 10 days, prior to homogenization by manual stirring. For each time collected, the baseline was corrected, assuming linear interpolation between the absorbances at 400 and 550 nm to estimate the value at 495 nm. A blank control experiment was carried out using bare chitin. Each condition was carried out in duplicate.

### 4.5. UV-Vis Spectroscopy and Confocal Microscopy

UV-Vis spectra of chitin samples were collected between 250 and 650 nm with a 1 nm resolution, and an average time of 0.1 s.

Confocal microscopy imaging was performed using an NIS-Elements C, Nikon, and exciting the sample using a 489.3 nm laser. The 512 × 512 images were acquired at a pixel size of 0.62 μm (optical resolution of 0.25 μm) and a Z step of 0.35 μm.

### 4.6. X-ray Diffraction and Infrared Spectroscopy

Attenuated total reflectance Fourier-transform infrared spectroscopy (ATR-FTIR) spectra were collected using a Nicolet IS10 spectrophotometer equipped with a germanium crystal ATR accessory. The spectra were obtained with 2 cm^−1^ resolution and 100 scans. Omnic software (Thermo Electron Corp., Woburn, MA) was used for data processing and baseline correction.

X-ray diffraction (XRD) patterns were collected using a PanAnalytical X’Pert Pro equipped with X’Celerator detector powder diffractometer using a Cu Kα radiation generated at 40 kV and 40 mA (λ = 1.54056 Å). The diffraction patterns were collected within the 2θ range from 4 ° to 25 ° with a step size (Δ2θ) of 0.05 ° and a counting time of 180 s. The chitin samples were analyzed by orienting the X-ray radiation plane parallel to the fibril orientation.

XRD and FTIR measurements were carried out in duplicate on two independent samples.

### 4.7. Scanning Electron Microscopy

SEM images were acquired with a Leica Cambridge Stereoscan 360 scanning electron microscope equipped with an Everhart and Thornley SE detector. The images were collected using a tension of 20 kV. For SEM, the wet specimens were lyophilized, eventually cut with a scalpel to expose the section, glued on carbon tape, dried overnight in a desiccator, and coated with 20 nm of gold layer prior to imaging them.

### 4.8. Uniaxial Tensile Tests

Monotonic uniaxial tensile tests were performed using a universal testing machine (mod. 4465 with Series IX software, Instron) and dedicated grips. Prior to analysis, each sample was hydrated in water for 24 h. While hydrated, each sample was cut in a proper dimension using scissors and tested. The actual width and thickness of each hydrated sample were measured before testing them using a SM-LUX POL microscope collecting images with a 5.0 MP digital camera (Motic Moticam 5+). The images were analyzed using ImageJ. Each sample was about 50–60 mm long, 6 mm wide, and 30–70 µm thick (the thickness varied between samples, because of the intrinsic variability of the initial biological samples). The samples were connected to the instrument, leaving a free length of about 40–50 mm between the clamps. The tests were performed with an actuator speed of 5 mm·min^−1^ (resulting in a strain rate of about 0.2%·sec^−1^) at room temperature. As the curves were rather linear until failure started, the following parameters could be calculated, taking into account the actual dimensions of each specimen:

- Young’s modulus of elasticity: defined as the slope of the linear part of the stress-strain curve (usually between 30% and 80% of the maximum strain) and calculated using linear interpolation (R^2^ ≥ 0.99). The portion of this region considered was reduced in case changes in the linearity were observed; in this case, only the initial linear region was considered;
- Maximum stress and strain.

At least five specimens were tested for each group. Slipping, early breakage, or inhomogeneity in data were not observed in the samples examined. Statistical analyses were performed using a *t*-test (ν = 8, *p* = 0.05).

## 5. Conclusions

A new route based on a one-step supramolecular functionalization of polysaccharide-based matrices in an aqueous solution is proposed. The idea builds upon the use of lectins, proteins with high affinities and specificities for polysaccharides, as linkers for functionalization. The validity of this new method was proved using WGA, a lectin, tagged with FITC, and a deproteinized squid pen, as a β-chitin hierarchically-organized matrix. This approach has several advantages over classical chemical functionalization: (i) a protein platform offers different chemical groups allowing an easy route for the functionalization of the material, especially when compared to a polysaccharide; (ii) the chemical functionalization of the protein occurs in the homo-phase, instead of the hetero-phase used for materials; (iii) lectins are strongly specific for polysaccharides, making the supramolecular functionalization homogeneous and highly selective; (iv) the polysaccharide supramolecular functionalization can occur in water and physiological environments. In addition, the separation of the lectin chemical functionalization and the polysaccharide supramolecular functionalization in two different steps allows the use of sensitive chemical moieties on the lectin without precluding the use of harsh treatments in the preparation of the polysaccharide matrix.

As proven in this study, the use of a supramolecular functionalization guarantees additional important benefits compared to a covalent interaction, i.e., it (i) does not alter the crystal structure of the target material; (ii) does not modify the texture or hierarchical organization of the matrix, even at the nanoscale; (iii) does not affect the interaction among polymer fibrils, leading to no changes in the material properties (i.e., mechanical properties). The complex formed appears very stable, with 93% of the lectin bound to the matrix after 10 days in solution. This slight loss of lectin might find important application in drug delivery (mainly due to the very slow-release kinetics). Moreover, since the functionalization can occur in a wide range of water-based environments, the matrix could be reloaded with the functionalized lectin. This allows an increase in the functionalization yield if necessary as well as the possibility to change the functionalization of the matrix directly in situ.

In conclusion, this methodology opens a landscape of possibilities in polysaccharide material science, especially in exploiting biogenic matrices, and can find application in almost every research field, including the medical field.

## Figures and Tables

**Table 1:** Crystallographic and mechanical data of the blank control and the WGA-treated samples. Diffraction peaks were indexed according to Gardner and Blackwell (1975) [77]; (010)/100) refers to a ratio between the intensities of the two peaks. Tensile tests were carried out using WGA not functionalized with FITC.

		Control			WGA		
XRD	(010)/°	8.3	±	0.2	8.1	±	0.1
	FWHM (010)/°	1.72	±	0.06	2.48	±	0.06
	(100)/°	19.7	±	0.1	19.72	±	0.03
	FWHM (100)/°	1.81	±	0.06	2.56	±	0.06
	(010)/(100)	0.75	±	0.09	0.69	±	0.04
Tensile tests	Max strain/%	1.3	±	0.4	2.0	±	0.8
	Max stress/MPa	87	±	23	95	±	32
	Young’s modulus/MPa	108	±	33	80	±	39

## Data Availability

Not applicable.

## Supplementary Materials

The following supporting information can be downloaded at: https://www.mdpi.com/article/10.3390/molecules27175633/s1, details on the synthesis and characterization of the WGA-FITC, and the WGA-FITC functionalized scaffold, the confocal analysis of the control sample, the desorption kinetic, the stress-strain curve of the mechanical tests, and the FTIR absorption band positions. Reference [73] is cited in the supplementary materials.
