# Supplementary material for: Supramolecular Binding with Lectins: A New Route for Non-Covalent Functionalization of Polysaccharide Matrices
Source: Molecules. 2022 Sep 1;27(17):5633. doi: 10.3390/molecules27175633 (PMC9457544; doi:10.3390/molecules27175633)
Supplement: Supplementary file 1 [file molecules-27-05633-s001.zip › molecules-1863625-supplementary.pdf]

Supporting Information

Lectins, a new route for non-covalent polysaccharide functionalization

Devis Montroni, Matteo Di Giosia, Matteo Calvaresi, and Giuseppe Falini*

Figure S1: UV-Vis spectrum of WGA-FITC p. 2

Figure S2: SDS-PAGE of WGA and WGA-FITC. P. 2

Figure S3: Confocal image of a control and WGA-FITC treated chitin sample sections. p. 3

Figure S4: Typical stress-strain plots from the uniaxial tensile tests. p. 3

Figure S5: Desorption kinetic of WGA-FITC in PBS. p. 4

Table S1: FTIR absorption bands reported in cm^{-1}. p. 5

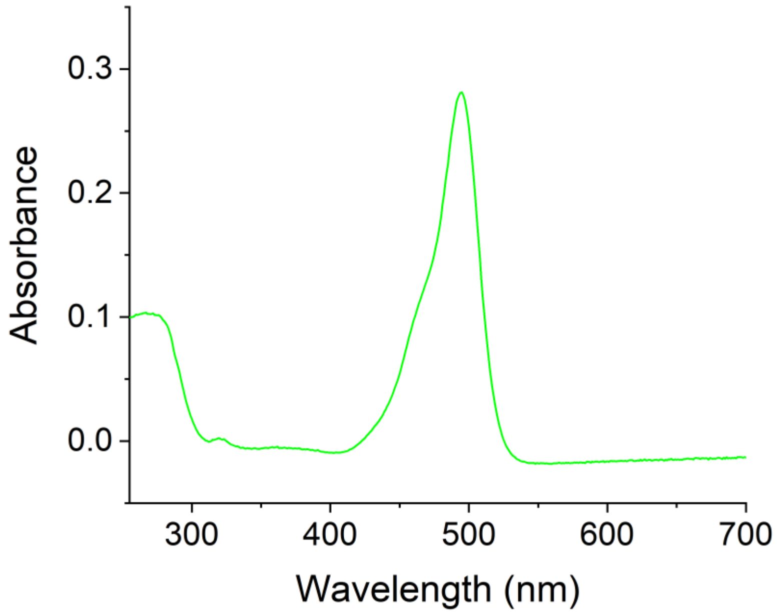

Figure S1: UV-Vis spectrum of WGA-FITC collected after dialysis purification. The absorption band, showing the maximum at 495 nm, is diagnostic for FITC and is used for FITC quantification.

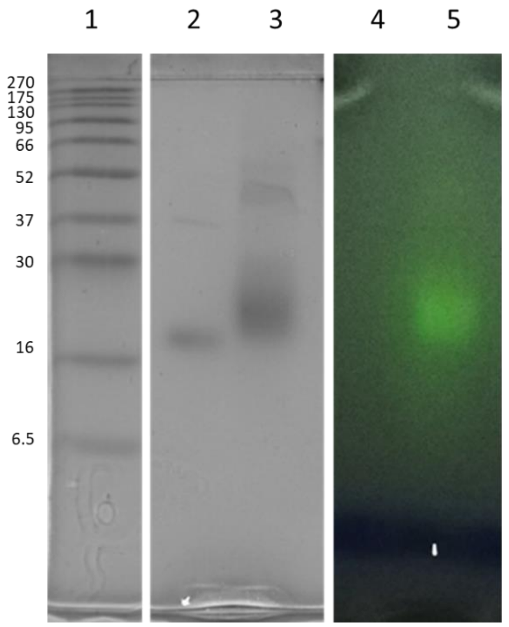

Figure S2. SDS-PAGE analysis of WGA and WGA-FITC. Each well was loaded with 10 μl of: 1) Elite Pre-stained Protein Ladder, 2) WGA, 3) WGA-FITC, acquired under white light illumination. 4) WGA, 5) WGA-FITC acquired under UV illumination (365 nm). Fluorescent imaging (lanes 4,5) was carried out before staining with Coomassie Blue G-250 (lanes 1,2,3).

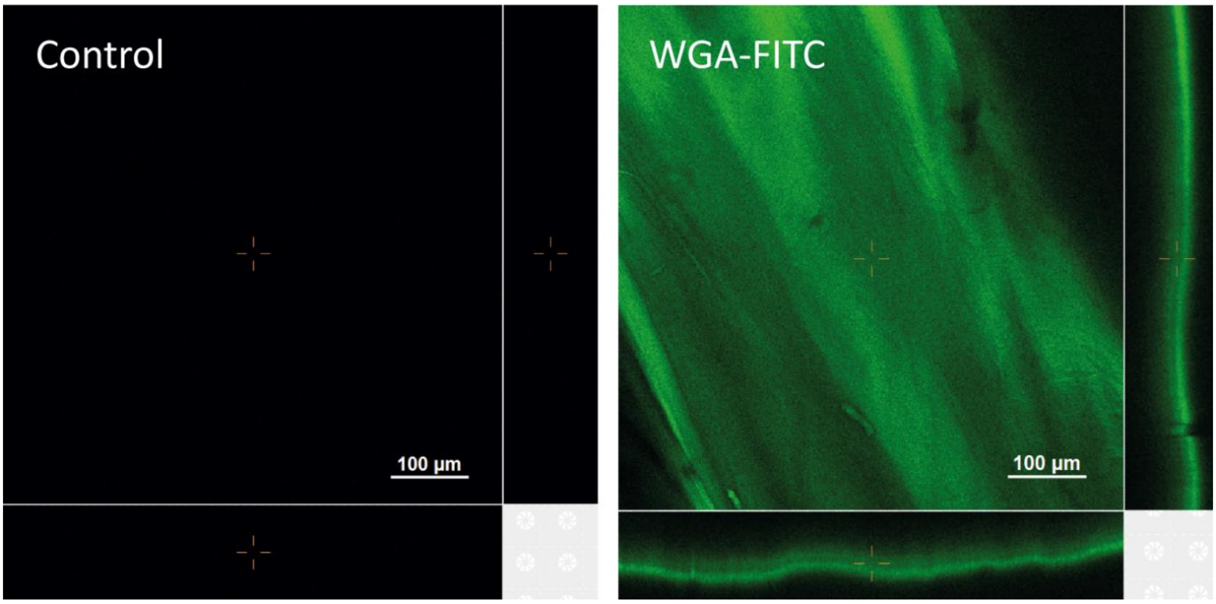

Figure S3: Confocal image of a control (left) and WGA-FITC treated (right) chitin samples.

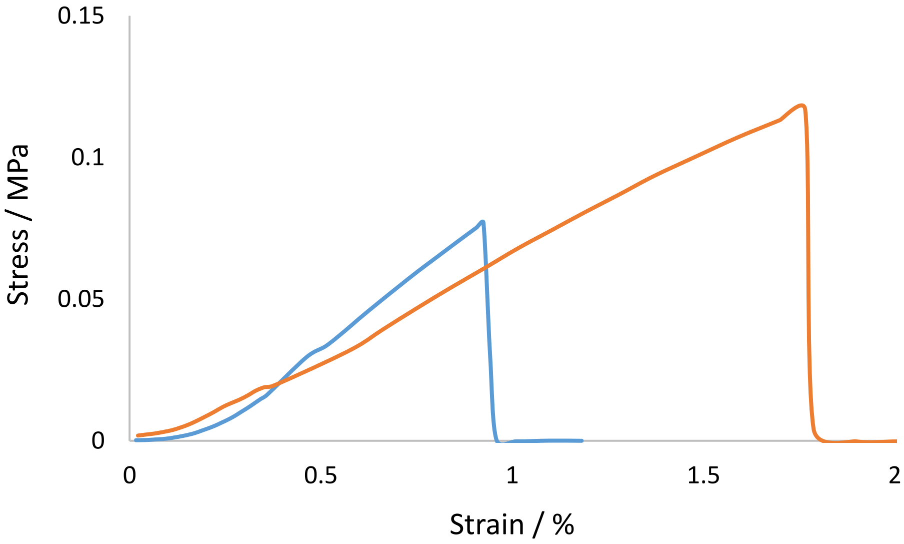

Figure S4: Typical stress-strain plots from the uniaxial tensile tests. One specimen for each group is shown, the WGA-treated matrix (orange) and the control matrix (blue).

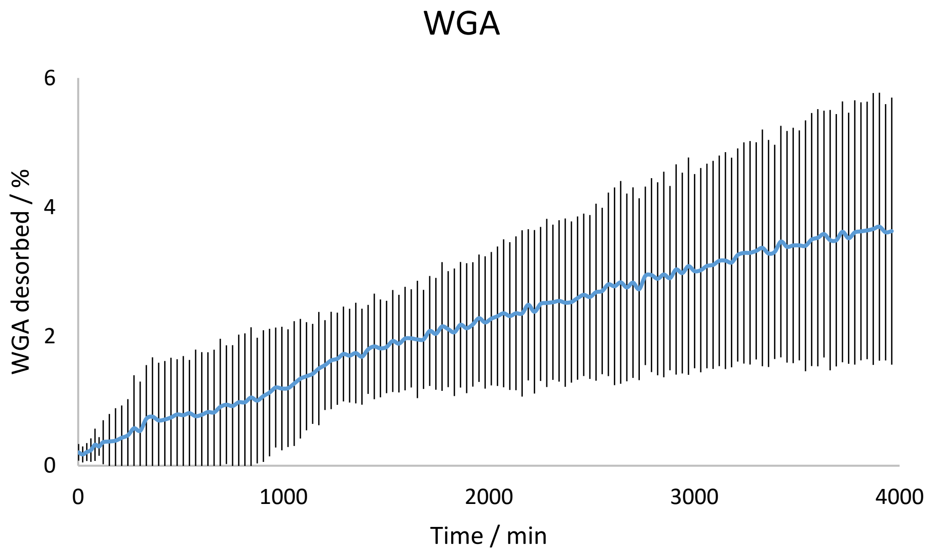

Figure S5: Desorption kinetic of WGA-FITC in PBS from the chitin sample expressed in percentage of WGA-FITC desorbed from the matrix respect to the one present in it.

Table S1: FTIR absorption bands reported in cm^{-1} (Pearson, Marchessault, & Liang, 1960).

Control	WGA-FITC	
3428	3424	OH stretching
3285	3279	NH stretching
3095	3098	NH stretching
2919	2823	CH_3 stretching
2868	2868	Symmetric CH_3 stretching and asymmetric CH_2 stretching
1655	1655	Amide I band
1554	1553	Amide II band
1431	1426	CH_2 bending and CH_3 deformation
1375	1375	CH bending and symmetric CH_3 deformation
1310	1308	CH_2 wagging
1262	1261	Amide III band
1202	1202	
1155	1155	Asymmetric bridge oxygen stretching
1112	1112	Asymmetric in-phase ring stretching mode
1068	1067	CO stretching
1031	1031	CO stretching
950	949	CH_3 wagging
905	900	Ring stretching

Pearson, F. G., Marchessault, R. H., & Liang, C. Y. (**1960**). Infrared spectra of crystalline polysaccharides. V. Chitin. *Journal of Polymer Science*, *43*(141), 101-116.
